# Species, sex and geographic variation in chlamydial prevalence in abundant wild Australian parrots

**DOI:** 10.1038/s41598-020-77500-5

**Published:** 2020-11-24

**Authors:** Helena S. Stokes, Johanne M. Martens, Ken Walder, Yonatan Segal, Mathew L. Berg, Andrew T. D. Bennett

**Affiliations:** 1 Centre for Integrative Ecology, School of Life and Environmental Sciences, Deakin University, 75 Pigdons Road, Waurn Ponds, VIC 3216 Australia; 2 Centre for Molecular and Medical Research, School of Medicine, Deakin University, 75 Pigdons Road, Waurn Ponds, VIC 3216 Australia; 3 Department of Jobs, Precincts and Regions, 475 Mickleham Road, Attwood, VIC 3049 Australia

**Keywords:** Ecological epidemiology, Conservation biology, Bacteria

## Abstract

*Chlamydia psittaci* (order: *Chlamydiales*) is a globally distributed zoonotic bacterium that can cause potentially fatal disease in birds and humans. Parrots are a major host, yet prevalence and risk factors for infection in wild parrots are largely unknown. Additionally, recent research suggests there is a diverse range of novel *Chlamydiales* circulating in wildlife. We therefore sampled seven abundant parrot species in south-eastern Australia, taking cloacal swabs and serum from n = 132 wild adults. We determined *C. psittaci* and *Chlamydiales* prevalence and seroprevalence, and tested for host species, sex, geographical and seasonal differences, and temporal changes in individual infection status. Across all species, *Chlamydiales* prevalence was 39.8% (95% CI 31.6, 48.7), *C. psittaci* prevalence was 9.8% (95% CI 5.7, 16.3) and *C. gallinacea* prevalence was 0.8% (95% CI 0.1, 4.5). Other *Chlamydiales* species were not identified to species level. We identified two *C. psittaci* strains within the 6BC clade, which is highly virulent in humans. Seroprevalence was 37.0% (95% CI 28.5, 46.4). Host species (including crimson rosellas, galahs, sulphur-crested cockatoos and blue-winged parrots) differed in seroprevalence and *Chlamydiales* prevalence. Galahs had both highest *Chlamydiales* prevalence (54.8%) and seroprevalence (74.1%). Seroprevalence differed between sites, with a larger difference in males (range 20–63%) than females (29–44%). We reveal a higher chlamydial prevalence than previously reported in many wild parrots, with implications for potential reservoirs, and transmission risks to humans and other avian hosts.

## Introduction

The majority of pathogens affecting animals have multiple host species, with many pathogens able to infect humans, domestic hosts and wildlife^1^. The importance of studying diseases and pathogens in wildlife populations has long been established, both for the sake of wildlife conservation and for effective management of pathogens that can infect humans and livestock^2^. Despite this, current pathogen surveillance in wildlife is limited and often ineffective, as it is primarily based on ‘passive’ surveillance, through sampling from dead or sick animals (often through community submissions) which are likely to be biased samples^3^. Several pathogens can be carried by wild animals without signs of clinical disease^4^, for which passive surveillance is likely to be particularly ineffective. The use of unbiased samples to analyse risk factors for infection in wildlife is a crucial step in identifying and modifying risk factors which favour the persistence of infection or likelihood of a disease outbreak, both in target wildlife species, and in livestock or humans, in the case of zoonotic pathogens^5^.

Bacteria in the order *Chlamydiales* are globally important pathogens. There are several hundred documented chlamydial hosts globally, the majority being wild mammals and birds^6^. *Chlamydia psittaci* is a well-documented zoonotic avian pathogen, with the capacity to cause severe disease and fatality in birds, humans and occasionally other mammals^7,8^. Other chlamydial species, such as *C. abortus* and *C. trachomatis*, have also been found in birds^9^, and three additional novel species and one Candidatus species of *Chlamydia* have been identified in avian hosts since 2013^10,11^. Birds are also a potential host for bacteria from other more recently described families in the order *Chlamydiales* (such as *Simkaniaceae* and *Parachlamydiaceae*) which may also cause disease^8^. Novel *Chlamydiales* species have recently been identified in sea birds, and mammals including ungulates and marsupials^8,12,13^, and they are likely to be found in many other wildlife populations, given the recent reported increase in chlamydial diversity in a range of hosts^6,8^.

Captive parrots (order Psittaciformes) have long been known to be major hosts of *C. psittaci*, with infected individuals reported worldwide^7,10,14,15^. Consequently, there is a global risk of zoonotic transmission to pet owners, veterinarians, zoo workers, and pet shop employees and visitors^10,16^. Surprisingly, however, there is little data on the prevalence of *C. psittaci* or other *Chlamydiales* in wild parrots, despite knowledge that *C. psittaci* can cause severe disease in parrots^17,^ one of the most highly threatened bird orders in the world^18^. Furthermore, *C. psittaci* strains in the 6BC clade, which is usually associated with parrots, are highly virulent in humans^19^. In countries where parrots are endemic or introduced, parrots may therefore be a reservoir for human infection, and the potentially severe disease this causes, ‘psittacosis’, which can result in pneumonia in up to 83% of cases^20,21^. *C. psittaci* was identified in early studies of wild Australian parrots^22,23^, and more recently in wild blue-fronted amazon parrots (*Amazona aestiva*) and hyacinth macaws (*Anodorhynchus hyacinthinus*) in Brazil^24^. *Chlamydiaceae* were also recently identified in feral ring-necked parakeets *(Psittacula krameri)* in France^25^. However, the study in Brazil only included nestlings, which may not be representative of the adult population, and the study in France only identified one *Chlamydiaceae* positive sample to species level. Evidence from captive birds shows considerable host variation in susceptibility^7^, so it is likely that some free-living species are more often infected than others. One early study of wild Australian parrots found 0% prevalence in galahs *(Eolophus roseicapillus*) compared to 18.3% and 12.5% prevalence in Adelaide rosellas *(Platycercus elegans adelaidae)* and red-rumped parrots (*Psephotus haematonotus),* respectively^23^. However, this study was carried out more than 60 years ago: prevalence may have changed, not least because wild parrot populations have shifted with changes in habitat and increased urbanisation^26^. While there have been more recent studies of *Chlamydia* prevalence in wild Australian parrots^27,28^, prevalence was not compared between host species, most likely because the prevalence was very low. Sex differences in chlamydial prevalence are also rarely investigated in birds, although physiological, ecological and behavioural differences between the sexes can drive differences in susceptibility and immunocompetence^29^, and incorporating knowledge of these differences is important when evaluating the impacts of infection on populations. In one recent study, all birds infected with *C. psittaci* were male, where the sex was known^28^. However, in most studies, including studies from other avian taxa, sex is either not recorded or the sample size is very small.

Analysis of swab samples (e.g. from the cloaca) by PCR is a commonly used method to detect *Chlamydia* in birds, because PCR assays are highly sensitive and easy to standardise across different studies^30^. However, as chlamydial shedding can be intermittent^7,31^, estimating chlamydial prevalence by this method alone may result in false negatives. Serological assays are not affected by sporadic shedding^32^, however many of these cannot distinguish between current and past infection^5^, and may have reduced sensitivity or specificity compared to molecular techniques^32^. Despite the values and limitations of both these assays, few studies use both molecular and serological techniques, although recent research suggests that using a single imperfect diagnostic method is likely to underestimate disease prevalence in wildlife^33^. Additionally, to our knowledge, there has been no repeated testing in wild birds for chlamydial infections, thus it is unknown how frequently or for how long individuals may shed *Chlamydia* into the environment and thereby present a transmission risk to conspecifics, or other host species. It is also unknown how long wild birds produce a detectable immune response, which may be different from findings in captive birds, particularly given that serologic responses vary widely between psittacine species^31^.

Parrots are widespread and abundant across Australia and found in a variety of habitats, including farmland, urban and peri-urban land^34^. Many species come into close contact with humans^26,35^. The most frequently observed species are galahs, sulphur-crested cockatoos *(Cacatua galerita),* crimson rosellas *(Platycercus elegans*) and rainbow lorikeets *(Trichoglossus moluccanus)*^36^. *C. psittaci* has been reported in some Australian parrot species^22,23,27^, however many reports are from captive birds, or case studies with a small sample size of each species. One early study reported 12% prevalence in wild Australian parrots^23^ (estimated primarily from galahs, crimson rosellas, and red-rumped parrots), but this figure should be taken with some caution, as some samples were simply pooled from multiple individuals, and the detection method used was not specific to *C. psittaci*. More recent prevalence estimates (derived from *C. psittaci*-specific PCR analysis) are lower, ranging between 0 and 1.8%^27,28,37^, although the exact subset and number of host species tested differ from the earlier study. However, one of the recent estimates was derived from passive surveillance, through sampling parrots brought into veterinary clinics^28^, and the other two studies only tested parrots from one family (Cacatuidae)^27,37^. The former prevalence estimate may thus be biased, and the latter two estimates may not be representative of other psittacine families.

Despite these low reported prevalences, wild Australian parrots are a hypothesised potential reservoir of human infection. A strain of *C. psittaci* known to be highly pathogenic for humans was isolated from a wild crimson rosella in New South Wales^19^ and direct contact with wild parrots was identified as a major risk factor for human infection in a community with endemic psittacosis^21^. Furthermore, previous data have shown that Australia has a higher number of human cases of psittacosis per capita than most other countries^38^. Signs of *C. psittaci* infection in wild Australian parrots are also little known, but as *C. psittaci* has been reported as a cause of acute illness and death in some psittacine species (including the orange-bellied parrot [*Neophema chrysogaster*]^39^ and several South American parrot species^17^) it is possible that some wild populations may be adversely affected, and that *C. psittaci* may pose a potential threat to parrot conservation. Reports of infected crimson rosellas range from emaciated to apparently healthy^19,22,40^ and infected galahs have been reported apathetic with diarrhoea^22^, but these are again case studies of individual birds, which are often concurrently infected with other pathogens^40^, making investigating the impacts of chlamydial infection alone challenging.

We investigated the prevalence of *C. psittaci* and related *Chlamydiales* in several psittacine species in south Victoria, Australia. We specifically selected four focal species: crimson rosellas, galahs, and sulphur-crested cockatoos (selected based on their abundance and widespread distribution) and blue-winged parrots *(Neophema chrystostoma)*, selected based on their ecological similarity with the critically endangered orange-bellied parrot, thus of conservation relevance. We aimed to determine (1) overall PCR prevalence and seroprevalence, (2) whether there were host species, sex or geographic differences in prevalence for our four focal species, (3) the relationship between infection and host body condition, and (4) how chlamydial infection status changes over time in recaptured individuals. Our goal was to improve knowledge about chlamydial prevalence in a wild parrot community, including risk factors for infection, and to identify whether these species may be a reservoir of chlamydial infection for other wild and domestic bird species, humans and other mammals.

## Results

### Identification and prevalence of Chlamydiales

Across all host individuals tested, and taking the first capture of every individual (Supplementary Tables S1–S2), mean total *Chlamydiales* PCR prevalence was 39.8% (95% CI 31.6, 48.7; 49/123 positive), *C. psittaci* PCR prevalence was 9.8% (95% CI 5.7, 16.3; 12/123 positive), and *C. gallinacea* PCR prevalence was 0.8% (95% CI 0.1, 4.5; 1/123 positive). Including all recaptures (n = 179), there was a total of n = 67 *Chlamydiales* positive sampling events. Of 14/67 samples positive for *C. psittaci,* n = 4 were identified by species-specific PCR, and n = 10 identified by sequencing, identified from BLASTn analysis using both the non-redundant nucleotide (nr/nt) database and 16S ribosomal RNA database (Table 1; Supplementary Table S3). One positive sample was identified as *C. gallinacea,* by using the *gidA* and CTU/CTL primers (individual reported in a previous study^41^, where *ompA* sequencing was carried out as confirmation). We sequenced 19 of the 52 remaining unknown *Chlamydiales* positive samples, of which n = 11 were successfully sequenced: n = 2 samples were identified as *Parachlamydiaceae,* n = 2 samples represent potentially novel species within the *Chlamydiales,* and n = 7 samples were mixed chlamydial infections, confirmed as *Chlamydiales* from BLASTn analysis using the nr/nt and 16S databases. Partial sequences of the *ompA* gene from two *C. psittaci* samples indicated that the *C. psittaci* genotypes grouped most closely with genotype A and the 6BC clade (Supplementary Figure S1). All 16S and *ompA* accession numbers obtained are provided in Table 1. Seroprevalence (according to the ImmunoComb assay) was 37.0% (95% CI 28.5, 46.4; 40/108 individuals were positive).

**Table 1:** Number of samples which tested positive for *Chlamydiales* by PCR, and sample identity as determined by species-specific PCR analyses and sequencing.

Species/Family	Number of positive samples identified	GenBank accessions
*C. psittaci*	14	MT356618–MT356622^a^, MT872000, MT872005, MT889682, MT889721, MT889722, MT875197, MT875198
*C. gallinacea*	1	MN114672^a^
*Parachlamydiaceae*	2	MT889690
Uncultured chlamydia-like bacteria	2	MT356624^a^
Mixed chlamydial infection	7	N/A
Unknown	41	N/A
TOTAL *Chlamydiales* PCR positive	67	

^a^Denotes sequences obtained in previous studies^41,42^.

### Testing for host species differences in prevalence

**Figure 1:**
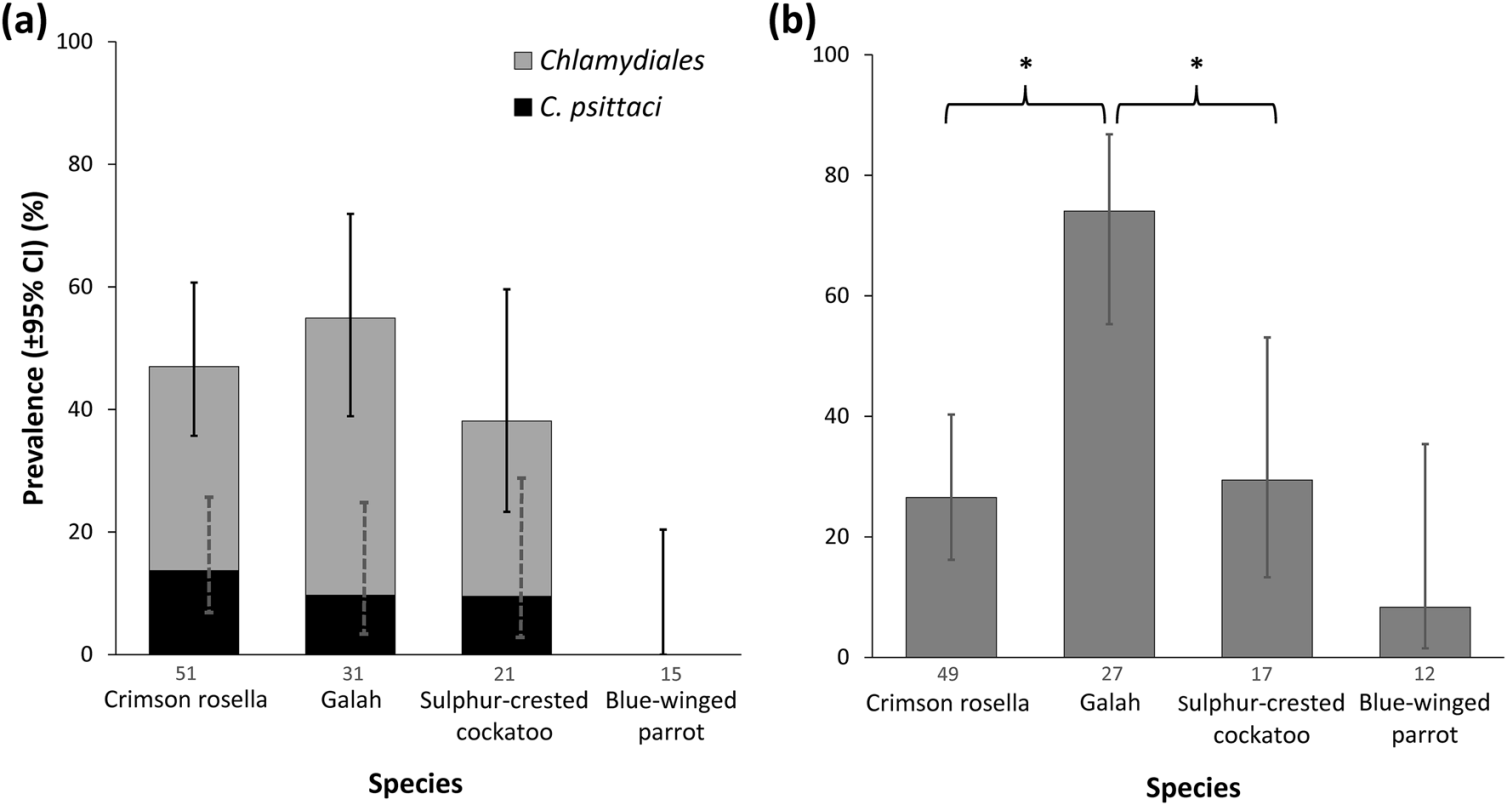
Comparison of prevalence and seroprevalence across host species: **(a)** *Chlamydiales* and *C. psittaci* PCR prevalence, and **(b)** seroprevalence. *denotes significant pairwise comparisons between species (where *p* < 0.05). Data labels indicate the sample size for each species.

*Chlamydiales*, including *C. psittaci*, were identified in all four focal host species except for blue-winged parrots (Fig. 1a). When analysing birds caught in walk-in traps only, we did not identify *C. psittaci* or *Chlamydiales* in the three species for which we had smaller sample size, namely eastern rosellas *(Platycercus eximius)* (n = 3), rainbow lorikeets (n = 2) and red-rumped parrots (n = 1). However, when including recapture data, we did find one breeding eastern rosella testing positive for *Chlamydiales*. Seropositive individuals were found for all four focal species (Fig. 1b). Of the remaining three species, one eastern rosella (33.3%; 95% CI 6.1, 79.2 [1/3 positive] assayed seropositive. Host species differed in *Chlamydiales* prevalence (*p* = 0.005) and seroprevalence (*p* < 0.001), but not in *C. psittaci* prevalence (Fig. 1, Table 2). Post-hoc pairwise comparisons revealed that galahs had significantly higher seroprevalence (74.1%; 95% CI 55.3, 86.8 [20/27 positive]) compared to crimson rosellas and sulphur-crested cockatoos (Fig. 1b, Supplementary Table S4). Galahs also had the highest *Chlamydiales* PCR prevalence (54.8%; 95% CI 37.8, 70.8 [17/31 positive]) but pairwise comparisons between host species were not significant.

**Table 2:** Associations between sex, species and field site and season, with *Chlamydiales* PCR prevalence, *C. psittaci* PCR prevalence and seroprevalence.

Response	Predictor	*χ*^2^	*df*	*p* value
*Chlamydiales* PCR prevalence (n = 117)	Sex	1.956	1	0.162
	Species	12.753	3	0.005*
	Field site	0.242	1	0.623
	Season	5.372	3	0.146
*C. psittaci* PCR prevalence (n = 117)	Sex	1.404	1	0.236
	Species	0.827	3	0.843
	Field site	0.007	1	0.932
	Season	1.524	3	0.677
Seroprevalence (n = 108)	Sex	0.869	1	0.351
	Species	26.813	3	< 0.001*
	Field site	4.748	1	0.029*
	Season	3.372	3	0.338
	Sex * Field site	4.161	1	0.041*

*denotes significance (*p* < 0.05).

### Testing for sex differences in prevalence

In females, *Chlamydiales* PCR prevalence was 51.1% (95% CI 37.2, 64.7; 24/47 individuals were positive) and *C. psittaci* prevalence was 14.9% (95% CI 7.4, 27.7; 7/47 individuals were positive). In males, *Chlamydiales* PCR prevalence was 33.3% (95% CI 23.7, 44.6; 25/75 positive) and *C. psittaci* prevalence was 6.7% (95% CI 2.9, 14.7; 5/75 positive). Conversely, male seroprevalence was 38.7% (95% CI 27.6, 51.2; 24/62 positive) and female seroprevalence was 34.8% (95% CI 22.7, 49.2; 16/46 positive). Sex differences in PCR prevalence or seroprevalence were not significant, however for seroprevalence there was a significant interaction between sex and field site (*p* = 0.041, Table 2).

### Testing for geographical and temporal differences in prevalence

**Figure 2:**
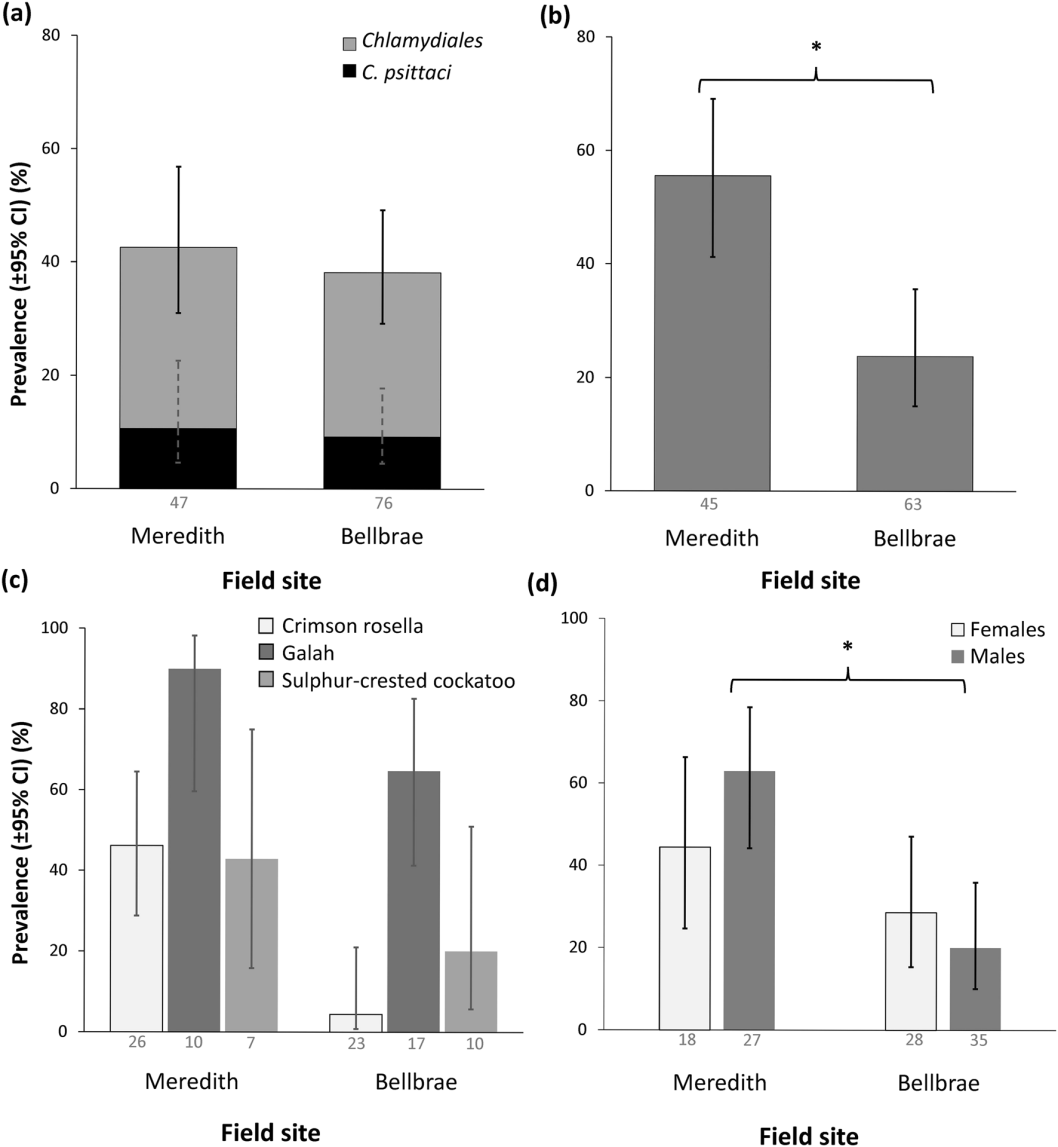
Comparison of prevalence and seroprevalence between field sites (Meredith and Bellbrae): **(a)** *Chlamydiales* and *C. psittaci* PCR prevalence; **(b)** seroprevalence; **(c)** seroprevalence for each species caught at both sites; and **(d)** seroprevalence for each sex. *denotes significance (*p* < 0.05). Data labels indicate the sample size for each group.

*Chlamydiales* prevalence at the Meredith site was 42.6% (95% CI 29.5, 56.7; 20/47 individuals were positive) and at the Bellbrae site was 38.2% (95% CI 28.1, 49.4; 29/76 individuals were positive) (Fig. 2a). *C. psittaci* prevalence at Meredith was 10.6% (95% CI 4.6, 22.6; 5/47 positive) and at Bellbrae was 9.2% (95% CI 4.5, 17.8; 7/76 positive). There were no significant differences between field sites for either *Chlamydiales* or *C. psittaci* PCR prevalence (Fig. 2a, Table 2). However, seroprevalence was more than twice as high at Meredith compared to Bellbrae (*p* = 0.029; Table 2; Fig. 2b). Meredith seroprevalence was 55.6% (95% CI 41.2, 69.1; 25/45 positive), compared to Bellbrae where seroprevalence was 23.4% (95% CI 14.7, 35.1; 15/64 positive). The pattern of higher seroprevalence at Meredith was observed in all three species caught at both locations (Fig. 2c). There was a significant interaction between sex and field site on seroprevalence, with a greater difference in male seroprevalence compared to female seroprevalence (*p* = 0.041, Table 2, Fig. 2d).

There was a trend for higher *Chlamydiales* prevalence in summer (83% [95% CI 43.6, 97.0; 5/6 individuals positive) and higher seroprevalence in autumn (67% [95% CI 46.7, 82.0; 16/24 positive). However, the effect of season on *Chlamydiales* prevalence, *C. psittaci* prevalence and seroprevalence was not significant (Table 2). Time of day had no effect on *Chlamydiales* or *C. psittaci* prevalence when all host species were tested. However, in crimson rosellas, birds were marginally more likely to test positive for *C. psittaci* in the morning compared to later in the day *(p* = 0.049; Supplementary Table S5).

### Relationship between host infection status and body condition

There was no effect of *Chlamydiales* or *C. psittaci* PCR prevalence, or seroprevalence, on body mass, packed cell volume (PCV; haematocrit) or residual body mass, although seropositive birds tended to have lower body mass (*p* = 0.051) and there was a tendency for *Chlamydiales* positive (*p* = 0.076) and seropositive (*p* = 0.071) birds to have lower PCV (Supplementary Tables S6–S7). There were species differences in PCV (*p* < 0.001), with galahs and blue-winged parrots having higher PCV compared to crimson rosellas and sulphur-crested cockatoos (Supplementary Table S8).

### Relationship between PCR prevalence and seroprevalence

Including recapture data, there were n = 138 capture events with valid assay results for both PCR and ImmunoComb. Of these 138 captures, there were n = 55 samples which assayed positive for *Chlamydiales* by PCR, of which we identified n = 19 samples to species or family level. There was a 65.7% agreement between results from the PCR and sequencing analysis and the ImmunoComb analysis (Supplementary Table S9). There was no significant relationship between *C. psittaci*, *Chlamydia* or *Chlamydiales* PCR prevalence and seroprevalence (Supplementary Table S10).

### Recaptures

There were n = 39 birds which were caught more than once (30 crimson rosellas, six galahs, and three eastern rosellas). Birds were caught a maximum of three times, and the mean interval between capture events was 214.2 days (± 132.0 SD, range 13–436; Supplementary Figure S2). *Chlamydiales* PCR status changed upon recapture in 41% of individuals, including one individual recaptured only 31 days later. Of the 14 birds where *Chlamydiales* status changed, 50% (7/14) assayed negative first then positive at recapture(s), 43% (6/14) assayed positive first then negative at recapture(s), and 7% (1/14) changed status twice (although only n = 5 birds were caught three times). Birds recaptured in a different season were more likely to change in *Chlamydiales* status compared to birds recaptured in the same season (*χ*^2^ = 3.904, *df* = 1, *p* = 0.048). In crimson rosellas*, Chlamydiales* status at first capture did not predict infection status at recapture (Supplementary Table S11).

Serostatus did not change in any birds tested for seroprevalence on multiple captures (Table 3). The mean interval between seropositive capture events was 78 days (± 46.8 SD, range 13–126) and the mean interval between seronegative capture events was 242.6 days (± 103.8 SD, range 14–436). Of four birds which assayed positive for *C. psittaci,* 75% (3/4) were seronegative, and did not assay positive for *C. psittaci* upon recapture (Table 3).

**Table 3:**
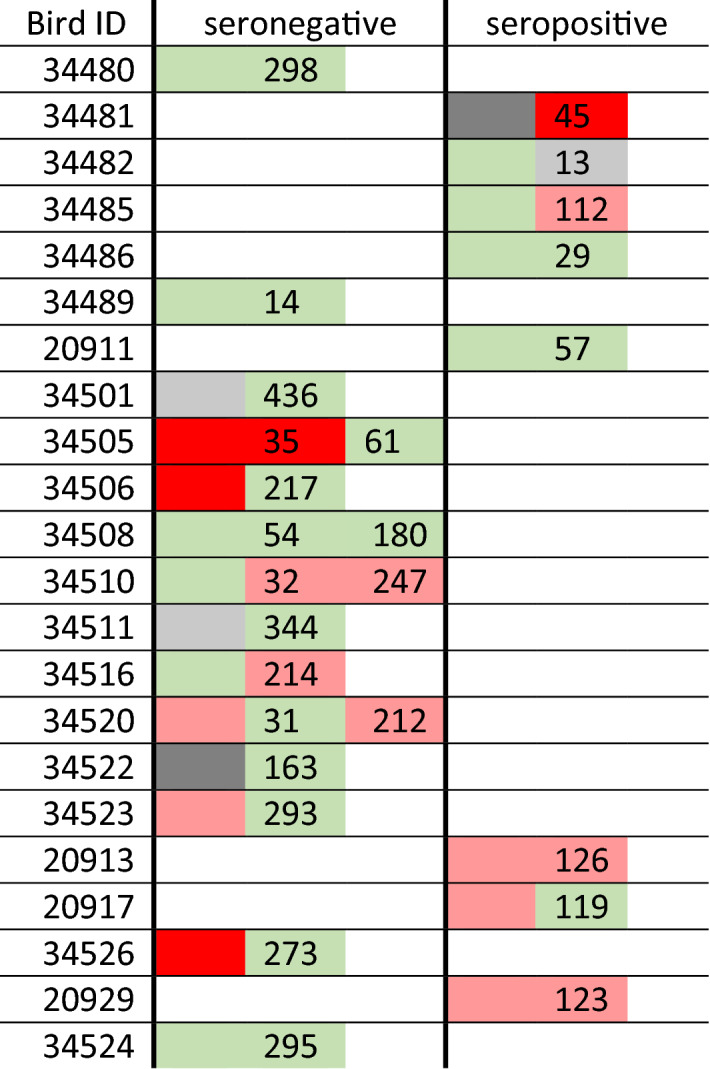
Serology and *Chlamydiales* infection status per individual for each capture, in all individuals with seroprevalence data for multiple capture events (n = 22).

The number indicates the number of days since the previous capture. Colour indicates chlamydial PCR test status: green is negative, pale red is *Chlamydiales* positive, bright red is *C. psittaci* positive, and dark grey is inconclusive. Light grey indicates captures where no cloacal swab was taken.

## Discussion

Prevalence data and risk factors for chlamydial infection in wild parrot populations are severely lacking, despite the disease risk these bacteria can pose to several avian hosts, and the potential risk of zoonotic transmission to humans. Furthermore, while *Chlamydiales* species outside the *Chlamydia* genus have been found in birds^12,43^, the overall prevalence of this bacterial order has rarely been investigated in a wild avian population or community. We identified 40% *Chlamydiales* prevalence in wild parrots, which is lower than that previously reported in wild mammals, namely marsupials (48%)^13^ and deer (72%)^44^. Our results provide further evidence that chlamydial infections are widespread in wildlife^6^. *Chlamydiales* were detected by PCR in crimson rosellas, galahs, sulphur-crested cockatoos and eastern rosellas, with the former three host species also testing positive for *C. psittaci.* At least two birds (a crimson rosella and a galah) were shedding *C. psittaci* strains from the 6BC clade, which is highly virulent for humans^19,45^. We also identified birds testing positive for *Parachlamydiaceae,* another *Chlamydiales* family identified as potentially pathogenic in humans^46^. Five host species were seropositive, namely crimson rosellas, galahs, sulphur-crested cockatoos, blue-winged parrots and eastern rosellas. It is plausible that the other host species we tested may also be infected with *C. psittaci* or other *Chlamydiales*, but our smaller sample size prevented us from detecting positive individuals. We found that host species differed in *Chlamydiales* prevalence, and that there were host species and geographical differences in seroprevalence.

Host species differences in *C. psittaci* prevalence have been reported in an early study of wild Australian parrots^23^, and in a more recent study of captive parrots^14^. Species differences in seroprevalence have been reported in captive parrots^15^. However, host species differences are rarely investigated in wild birds. We found that host species predicted *Chlamydiales* PCR prevalence, although the host species in which we did find infections did not differ significantly from each other in prevalence. We found no significant association between host species and *C. psittaci* prevalence, although this may be due to low statistical power, given the low *C. psittaci* prevalence in our sample. We found that host species predicted seroprevalence, with galahs having significantly higher seroprevalence compared to crimson rosellas and sulphur-crested cockatoos, suggesting that galahs may have a higher level of exposure to *Chlamydia* compared with the other host species. We also identified a higher *C. psittaci* prevalence in galahs (10%) than previously reported in this host species, with previous estimates between 0 and 2%^23,27,28^. Ecological or behavioural differences may result in increased chlamydial exposure in certain hosts. For instance, galah foraging behaviour may facilitate higher rates of infection: they typically forage on the ground^47,48^ which may cause them to become infected more frequently, as *C. psittaci* is transmissible through infected fomites^38^ and other *Chlamydiales* are also hypothesised to be transmitted through environmental contamination^49^. Interestingly, we also identified one galah infected with *C. gallinacea*^41^, a chlamydial species more frequently associated with poultry^10^. This could suggest a potential route of chlamydial transmission between wild parrots and free-range poultry (which may be bidirectional), and may warrant increased biosecurity measures on farmland. While seroprevalence was lower in crimson rosellas and sulphur-crested cockatoos, we also identified *C. psittaci* in these species, at 14% and 10% prevalence respectively. *C. psittaci* has previously been reported in wild crimson rosellas^19,28^, but in the few studies we found of wild sulphur-crested cockatoos, no birds assayed positive for *C. psittaci*^27,37^, except for one individual concurrently infected with beak and feather disease virus (BFDV)^28^. We found no blue-winged parrots shedding either *C. psittaci* or *Chlamydiales*, and only one individual which tested seropositive. This may indicate that they are less susceptible to infection, or that infected birds suffer severe disease or fatality prior to detection. The latter may be more likely given that parrots in the *Neophema* genus are reported as hard to treat for *C. psittaci* infection^7^ and *C. psittaci* has previously caused fatality in captive populations of the closely-related orange-bellied parrot^39^. To our knowledge, no other studies have tested wild blue-winged parrots for chlamydial infection.

We found geographic variation in seroprevalence, with a significant difference between field sites. However, we found no geographic variation in PCR prevalence. Consequently, our findings are more likely to indicate a previous high infection rate or outbreak in Meredith than a current high infection rate, and suggest that chlamydial exposure varies by location, over a relatively local scale. It is possible that this site variation in seroprevalence may partly be due to seasonal variation. However, we think this is unlikely, as IgG antibodies can persist in the host for several months following infection^50^. Moreover, our data shows that seroprevalence status did not change in birds recaptured several months later, suggesting that sampling date is unlikely to bias our seroprevalence data. Geographic variation in seroprevalence could arise from differences in site environmental characteristics, or bird community composition. Food availability, altitude and other habitat characteristics can predict malarial parasitaemia in wild birds^51^, and variation in bird community composition is suggested to cause geographic variation in *Mycoplasma gallisepticum* prevalence in house finches *(Carpodacus mexicanus)*^52^. Indeed, *C. psittaci* prevalence in pigeons in Europe has been shown to range between 16 and 28% across comparable distances to those separating our field sites (< 100 km), as well as differing between lofts in the same city^53^. The geographic variation we observed may mean that in certain locations there is a greater risk of chlamydial disease outbreak in birds, and consequently a greater risk of transmission to humans and livestock. Serological surveillance could be carried out in wild birds found in close proximity to livestock or human communities, to investigate these risks. Interestingly, while we did not find significant sex differences in overall PCR prevalence or seroprevalence, we found that the site differences we observed in seroprevalence were greater in males than in females. It is possible that a previous outbreak occurred in Meredith, and males were more susceptible to chlamydial infection than females, since males are more susceptible to infection in most vertebrate species^29^. Alternatively, as inherent sex differences in immune response and antibody persistence may occur^29,54^, it is possible that males have a longer-lasting antibody response. Therefore, if there was an outbreak at this site, male seroprevalence would remain elevated for a longer time. To our knowledge, no studies have tested for sex differences in chlamydial prevalence or seroprevalence in any wild bird species. Previous studies from captive parrots have shown either no significant sex differences in prevalence^55^, or in contrast to our findings, a higher seroprevalence in females^15^.

The overall *C. psittaci* prevalence we found (10%) was higher than that reported in other recent wild parrot studies in Australia and worldwide^27,28,56^, which could be due to a number of reasons. Firstly, as discussed, there is likely to be geographical variation. Secondly, sampling time of year may also cause variation in prevalence. For instance, we found a high *Chlamydiales* prevalence during summer (83%) which could be due to increased shedding due to the stress of breeding or moulting^34,38,57^. Additionally, estimated prevalence may vary between studies because different chlamydial PCR assays vary in sensitivity^30^. The 16SIG PCR assay and detection method we used appears to be very sensitive, because (a) we identified some 16S positive samples which could not be further characterised by sequencing, and (b) some *C. psittaci* positive samples (identified by sequencing) tested positive using the 16SIG PCR, but not the *C. psittaci* specific PCR. However, pan-*Chlamydiales* PCR primers such as those we used can also have lower specificity than other nested PCR or qPCR methods^58^. Consequently, it is possible we identified some false positives, which could have resulted in an overestimation of chlamydial prevalence. The *C. psittaci* positive samples which we only identified through 16S sequencing may have a low bacterial load, representing low-level infections; in future studies, using an additional PCR (e.g. one targeting another gene) may help to confirm sequencing results for *C. psittaci* identification. Other studies have also shown that samples with low *C. psittaci* loads may not be amplified by all PCR protocols^59^, or may only be identified by sequencing^27^. It is plausible these low-level infections have little effect on the host, and may not be of zoonotic risk: whether this is the case remains to be determined.

A limitation of our study is that we did not characterise the genotype of all *C. psittaci* positive samples. In future, it would be useful to characterise the genotype of all *C. psittaci* strains found, to facilitate comparison of strains between and across host species, to help identify potential transmission pathways, and to quantify zoonotic transmission risks. Nonetheless, because all *C. psittaci* strains are considered transmissible to humans^10,60^ and we identified at least two individuals shedding *C. psittaci* strains in a clade highly virulent in humans, we consider our findings are of zoonotic relevance. Another limitation is that some of the samples we sequenced were unsuccessful, most likely due to mixed infections, or low DNA concentration. Additionally, we did not sequence all PCR positive samples, so it is possible we may have detected bacteria outside the order *Chlamydiales;* however we consider this unlikely, since results from this study and our previous work^42^ confirmed that all successfully sequenced samples were within the *Chlamydiales*. The short fragment (298 bp) also prevented us from identifying all sequenced *Chlamydiales* to species level; future studies could use primers targeting a larger fragment, to facilitate identification to a genus or species.

Our PCR data from recaptured individuals showed that 41% of recaptured birds assayed differently between captures. Similar results were found for BFDV infection in crimson rosellas, where 77% of individuals which tested BFDV positive at least once tested differently upon recapture^61^. We found birds which assayed seropositive on both capture events up to four months apart, and we also identified seropositive birds which always assayed *C. psittaci* and *Chlamydiales* PCR negative. These could be chronically infected individuals which shed *Chlamydia* intermittently, as commonly found in captive parrots^7,38^. If this is occurring, it increases the risk of transmission to conspecifics, and to other species, in cases where different species share habitat or nesting hollows^62^. Our results could also be explained by sporadic infection and re-infection, infection relapses, or recovered birds assaying seropositive due to antibody persistence^50^. Re-exposure could cause longer-lasting antibodies and a boosted immune response, as hypothesised for avian influenza antibodies in recaptured waterbirds^63^. It is also possible that chlamydial shedding may follow a circadian rhythm. Indeed, in crimson rosellas at least, we found more birds testing positive for *C. psittaci* in the morning. To our knowledge, circadian variation in chlamydial shedding has not received prior investigation. Consequently, this may warrant further investigation, particularly as such effects may influence detectability and repeatability of chlamydial testing. To investigate whether multiple infections, chronic latent infection, or intermittent shedding is occurring, future studies could test whether recaptured birds are always infected with the same or different chlamydial strains, and test known chlamydial positive birds in captivity periodically throughout the day. Our data also suggest that chlamydial exposure is not ubiquitous in wild populations, as we identified individuals which consistently assayed seronegative at capture events more than a year apart, suggesting that birds may not be exposed to *Chlamydia* for several months at a time. We also identified seronegative birds which assayed PCR positive for *C. psittaci*. These birds may be in an early stage of infection, and not yet producing a detectable immune response^50^, or alternatively, they may have had low-level infections, which did not induce an immune response, since the infectious dose of a pathogen can affect host antibody response^64^. Two birds tested *C. psittaci* positive on initial capture, but seronegative upon their recaptures several months later; it is possible these birds seroconverted after initial capture, then stopped producing IgG antibodies following recovery^65^. A limitation is that we do not know the precise sensitivity or specificity of the ImmunoComb. It is plausible that this assay (designed to detect *C. psittaci)* also detects antibodies against other chlamydial species^41^, and cross reactivity to other bacteria may also occur^66^. Future studies could develop species-specific peptide based ELISAs to test for exposure to each chlamydial species; such an approach has been found to have increased specificity for *C. abortus* detection in livestock^66^, and could similarly increase the reliability of seroprevalence estimates for wild birds. Interestingly, we found no relationship between seroprevalence status and any of the combinations of PCR status tested. This lack of relationship (and our recapture findings) indicate that neither PCR nor serology alone can confirm the presence or absence of chlamydial infection in a population. Similar results were found for feline foamy virus infection in wild pumas *(Puma concolor)* where ELISA and qPCR did not have strong diagnostic agreement^33^. We suggest that using both PCR and serology is desirable for accurate estimation of chlamydial prevalence, and epidemiological inference.

We found no effect of chlamydial infection on host body condition, which accords with our recent study of crimson rosellas^42^. This could be indicative of endemic infection, whereby wild parrots have a stable host-parasite relationship with *Chlamydiales*, as similarly hypothesised by de Freitas Raso et al. for hyacinth macaws in Brazil^24^. The infections we observed may have a low bacterial load, or may be of low virulence. Indeed, *C. psittaci* genotype A (which we identified in two individuals) is endemic among captive psittacine birds^10^, so under natural conditions, they may suffer few adverse consequences of chlamydial infection. However, we did find that seropositive birds tended to have lower body mass and PCV, suggesting that there may be a link between infection and host body condition. This would be a useful area for further study in both captive and wild individuals.

In conclusion, we show that wild individuals of common parrot species in south-eastern Australia are both exposed to, and shedding, *C. psittaci* and other *Chlamydiales*, at a higher prevalence than previously reported in most wild parrot populations. For the first time in wild parrots, we demonstrate that host species within a community differ in *Chlamydiales* prevalence, and that seroprevalence differs between host species, and for males at least, geographical location. We also reveal that some individuals show evidence of antibody persistence and potentially chronic infection, which has implications for direct and environmental transmission. Highlighting the wide range and abundance of potentially zoonotic chlamydial bacteria in wild birds, our findings suggest that conservation managers should investigate the presence of these bacteria when managing threatened species, and investigate the potential spill-over risks in locations where humans and livestock are in contact with wild birds.

## Methods

### Sample collection

From 12 April 2017 until 31 October 2018, n = 132 adult wild parrots were captured and sampled, with n = 39 birds caught more than once (Supplementary Table S1). We selected four focal host species to investigate risk factors for infection; namely crimson rosellas [n = 57], galahs [n = 31], sulphur-crested cockatoos [n = 21]*,* and blue-winged parrots [n = 17]. The remaining three parrot species caught were eastern rosellas [n = 3], rainbow lorikeets [n = 2] and a red-rumped parrot [n = 1]. Parrots were captured in two study areas in south Victoria, Australia: either within 10 km of Bellbrae (S38°19′ E144°10′) or within 12 km of Meredith (S37°51′ E144°06′). These areas are located approximately 75 km apart, and we therefore considered these two different parrot communities, as previous data indicate that most recaptured crimson rosellas were caught or resighted within 10 km of their banding site^67^, and galahs and sulphur-crested cockatoos within 20 km of their banding site^34,35,68^. Birds were caught using walk-in traps and mist nets. Upon capture, each bird was placed in a bag and weighed. Following this, each bird was banded, where possible the wing, head-bill, tail and tarsus length was measured, and blood and cloacal swab samples taken. Blood was collected from the brachial vein^69^, stored at 4 °C immediately after collection, then centrifuged for 9 min at 16,000*g* within 3 h of collection, after which serum was separated using a Hamilton syringe and stored at − 80 °C. Cloacal swabs were stored at 4 °C immediately after collection, then stored at − 80 °C within 12 h of collection. PCV was measured as described by Ots, Murumägi & Hõrak^70^.

### DNA extraction and sequence analysis

DNA was extracted from cloacal swabs using an ammonium acetate extraction method^71^ modified for swabs, with a no-template control sample included in each batch. To summarise, swabs were placed into 250 µl of Digsol buffer (20 mM EDTA, 120 mM NaCl, 50 mM Tris–HCl, 20% SDS) with 10 µl of Proteinase K (10 mg/ml). Samples were digested overnight (minimum 15 h) at 37 °C, and following this 300 µl of 4 M ammonium acetate was added. 100% ethanol was added to precipitate the DNA, after which each sample was washed with 70% ethanol and re-suspended in low Tris–EDTA buffer (10 mM Tris–HCl, 0.1 mM EDTA, pH 7.5–8.0). DNA quantity was verified using a NanoDrop 1000 spectrophotometer, and prior to PCR analysis, samples with a DNA concentration of  > 50 ng/µl were diluted to 50 ng/µl. A multi-step PCR protocol was used to determine which chlamydial species were present in samples. DNA samples were firstly assayed for the presence of *Chlamydiales* using the pan-*Chlamydiales* 16SIG PCR^72^ (Table 4). Following this, positive samples were assayed using two separate species-specific PCR assays, using the *C. psittaci-*specific F3/B3 primers^73^ and the *C. gallinacea-*specific *gid*A primers^74^ to identify whether *C. psittaci* or *C. gallinacea* was present (Table 4). The 16SIG reaction was performed in 50 µl total reaction volume, containing 2 µl of extracted DNA, 5 µl of each 10 µM primer, 5 µl each of 10× buffer and dNTPs, 3 µl of MgSO_4_ and 1 µl of KOD Hot Start Polymerase (Novagen). Cycling conditions were as follows: initial denaturing period of 10 min at 95 °C, then 35 cycles of 1 min at 94 °C, 30 s at 68 °C and 1 min at 72 °C, followed by a final extension period of 7 min at 72 °C. Reaction conditions were the same for both the F3/B3 and *gid*A primers, with annealing temperatures of 57 °C and 59 °C respectively. Positive controls included a dilution of *C. psittaci* DNA for the 16SIG primers, DNA from a known *C. psittaci* positive bird for the F3/B3 primers, and a dilution of *C. gallinacea* DNA for the *gidA* primers. All negative controls were nuclease free water. All reactions were carried out in a GeneAmp PCR System 9700 thermocycler (Applied Biosystems, California U.S.A.). PCR product was visualised on a 1.5% Agarose gel, using 0.5× Tris–Borate EDTA buffer and SYBR Safe DNA Gel Stain (Invitrogen). Agarose gels were viewed under UV light and analysed using ImageLab 6.0.1 (Bio-Rad, California, U.S.A.). Samples with bands of intensity ≥ 5% of that of the positive control were considered positive. Where samples produced multiple bands or smears, they were re-assayed by PCR. Samples (n = 4) which did not produce a clear single band following re-analysis were considered ‘inconclusive’ as they were deemed of insufficient quality for further PCR or sequencing analysis.

**Table 4:** Oligonucleotide primers used to determine chlamydial prevalence and *C. psittaci* genotype.

Primer	Specificity	Target	Sequence (5′–3′)	Size (bp)	References
16SIG	All *Chlamydiales*	16S rRNA	F: CGGCGTGGATGAGGCAT R: TCAGTCCCAGTGTTGGC	298	Everett et al.^72^
F3/B3	*C. psittaci*	*Cpsit*_0607	F: AGAACCGGATTAGGAGTCTT R: GCTGCTAAAGCGAGTATTGA	263	Jelocnik et al.^73^
*gid*A	*C. gallinacea*	*gid*A	F: TTTATCATTAAAACAGCGTGGTTTC R: GAGGTGGCGATCTTTTTCAGAG	331	Li et al.^74^
CTU/CTL	*C. psittaci* (and *C. gallinacea*)^41^	*ompA*	F: ATGAAAAAACTCTTGAAATCGG R: CCAGCTTTTCTAGACTTCATCTTGTT	1070	Denamur et al.^77^

A subset of samples (n = 29) which assayed positive using the 16SIG PCR but negative for the two species-specific PCR protocols were sequenced using Sanger sequencing, to further interrogate the genetic identity of the amplicon. The amplified product from the 16SIG PCR was purified, then underwent a Big-Dye terminator reaction and dual-direction Sanger sequencing at the Australian Genome Research Facility (Melbourne). Sample chromatograms were analysed using MEGA X^75^, and sequences were compared against the nr/nt database using the BLASTn tool^76^. Samples were classed as *C. psittaci* if the top 10 BLAST hits had > 99% nucleotide identity and 99–100% query cover length with previously described *C. psittaci* 16S sequences, and E values of < 0.00001 (Supplementary Table S3). Similarly, samples with top BLAST hits with > 85% similarity with other published *Chlamydiales* sequences and above parameters in the nr/nt database were classified to family level or listed as ‘other uncultured bacteria’. Chromatograms with multiple double peaks but > 90% percentage similarity with *Chlamydiales* (n = 7) were classed as mixed infections. Samples which were not successfully sequenced or had chromatograms of bad quality (n = 8) were classed as inconclusive. For two *C. psittaci* positive samples with high DNA concentration and band intensity (from one crimson rosella, and one galah), we used the CTU and CTL primers^77^ to amplify an approximately 1070 bp fragment of the *ompA* gene, to investigate which genotypes are present (Table 4). Sequences were aligned with other publicly available *ompA* sequences in GenBank in MEGA-X^75^, using ClustalW. The *ompA* product of the positive *C. gallinacea* sample was also sequenced, the results of which are reported in a separate study^41^. All sequences are deposited in GenBank (see Table 1 for accession numbers).

### Serological analysis

Serum samples (n = 108) were assayed for antibody presence using the ImmunoComb solid-phase ELISA (Biogal, Kibbutz Galed, Israel). This kit has been validated for use in rosellas and cockatoos^78^ and a wide range of other psittacine and non-psittacine birds^78^. In brief, each serum sample corresponds with a colour change, with the colour intensity indicating whether or not antibodies are present^79^. Samples were allocated a colour intensity score from 0 to 5.5 in increments of 0.5, and compared for intensity against a positive control, which had a score of 3. Samples with scores of 0 were classed as negative, samples with scores of ≥ 2.5 were classed as positive, and samples with scores of 1–2 (n = 18) classed as inconclusive^79^. Inconclusive samples were excluded from analysis. A negative control was included in every reaction. The reliability of this method was confirmed by carrying out repeated scoring analysis of a subset of samples (n = 40) in a previous study^42^.

### Statistical analysis

Statistical analyses were carried out in R^80^. Prevalence values are reported with 95% confidence intervals, using the Wilson score interval. For our four focal host species we used Generalized Linear Models (GLM) to test associations between prevalence and host species, sex, field site, and season, with *Chlamydiales* PCR prevalence, *C. psittaci* PCR prevalence and seroprevalence each modelled as a binary logistic response in separate GLMs. *C. gallinacea* PCR prevalence was too low to analyse this prevalence independently. Sex, host species, season and field site were included as fixed effects in each model. Seasons were defined as follows: autumn: 1 March to 31 May; winter: 1 June to 31 August; spring 1 September to 30 November; summer: 1 December to 28 February. To deal with data separation in PCR prevalence, Firth's penalized maximum likelihood method was used^81^ by implementing the logistf package^82^ in R. Results were summarised using likelihood ratio test values (or penalized likelihood ratio test values for penalized maximum likelihood estimates) and associated *p*-values. We also tested all two-way and three-way interactions between model terms in addition to main effects, but interactions were only retained in the final models when significant (*p* < 0.05). Only one two-way interaction was retained (Table 2) as all other interactions had a *p*-value > 0.1. Post-hoc Tukey tests were used to estimate pairwise differences between species. We also ran GLMs for PCR prevalence with ‘time caught’ included as an additional fixed factor, to test whether prevalence varied between different times of day. We tested this first for all focal species, and then for crimson rosellas separately, as the host species with largest sample size and most capture time variation.

For focal species with positive and negative birds (crimson rosellas, galahs and sulphur-crested cockatoos, plus blue-winged parrots for seroprevalence), we used GLMs to test for an effect of *Chlamydiales* prevalence, *C. psittaci* prevalence and seroprevalence on raw body mass and PCV. We used body mass as this is a reliable measure of body fat content^83^ and we used PCV as this is a physiological measure often affected by disease or other environmental stressors^84^. We controlled for host sex and species, as these are strong predictors of body mass and can also cause variation in haematocrit^84^. For crimson rosellas and galahs (the host species with more morphometric data), we also carried out analyses using residual body mass (from regression of body mass on tarsus length)^85^ as an additional measure of condition, to control for size differences^70,85^. Residual body mass was calculated separately for each species. Each condition index was modelled as a linear response. Mean PCV for each species (± SD) is reported in Supplementary Table S8.

We report the level of diagnostic agreement between our PCR analysis and seroprevalence analysis, based on the assumption that the ImmunoComb detects antibodies against both *C. psittaci* and *C. gallinacea*^41^. We tested the relationship between PCR and seroprevalence by using separate GLMs to investigate whether seroprevalence (binary logistic response) was predicted by: [1] *C. psittaci* PCR status, [2] *Chlamydia* (genus) PCR status, and [3] *Chlamydiales* PCR status. For these analyses, we excluded *Chlamydiales* positive samples where the bacterial species was unknown.

For all prevalence analyses described above, we only analysed data from the first capture of each individual bird. For analysis of individual changes in infection status, we included data from recapture events, including recapture data from breeding birds (n = 16) which were caught in nest box traps as part of a separate study. We first used a GLM to test whether birds recaptured in different seasons were more likely to change in PCR status, with ‘change in PCR status (Y/N)’ modelled as a binary response, and ‘caught in different season (Y/N)’ as a predictor. For crimson rosellas*,* we used a binary logistic regression to investigate whether *Chlamydiales* infection status at first capture (positive or negative, according to PCR) predicted *Chlamydiales* status at recapture. We repeated this analysis excluding individuals (n = 2) which had a recapture interval of less than 4 weeks, to account for the possibility that recaptures after very short periods of time would not reveal any biologically relevant changes in infection status. We also ran these analyses including ‘number of days between capture’ as a covariate, to account for the likelihood that individuals recaptured at shorter intervals were more likely to assay the same upon recapture.

### Ethical statement

All sampling for this study was approved by the Animal Ethics Committee of Deakin University (permit B31-2015) and carried out under ABBBS banding authority 2319. All handling and use of animals conforms to the *Australian Code of Practice for the Care and Use of Animals for Scientific Purposes.*

## Supplementary information

Supplementary Information.

Supplementary Information.

## Data Availability

Data available from corresponding author upon reasonable request.
