# Supplementary material for: Species, sex and geographic variation in chlamydial prevalence in abundant wild Australian parrots
Source: Sci Rep. 2020 Nov 24;10:20478. doi: 10.1038/s41598-020-77500-5 (PMC7686501; doi:10.1038/s41598-020-77500-5)
Supplement: Supplementary file 1 — Supplementary Information. [file 41598_2020_77500_MOESM1_ESM.docx]

**Supplementary material**

**Species, sex and geographic variation in chlamydial prevalence in abundant wild Australian parrots**

Helena S. Stokes^1^*, Johanne M. Martens^1^, Ken Walder^2^, Yonatan Segal^3^, Mathew L. Berg^1^, Andrew T. D. Bennett^1^

*^1^ School of Life and Environmental Sciences, Centre for Integrative Ecology, Deakin University, 75 Pigdons Road, Waurn Ponds VIC 3216, Australia*

*^2^Centre for Molecular and Medical Research, School of Medicine, Deakin University, 75 Pigdons Road, Waurn Ponds VIC 3216, Australia*

*^3^Department of Jobs, Precincts and Regions, 475 Mickleham Road, Attwood VIC 3049, Australia*

*corresponding author, hsstokes@deakin.edu.au

**Supplementary Table S1** Sampling and chlamydial test data for all birds caught in this study *[see Excel file]*

**Supplementary Table S2** Total number of birds of each species caught, per season.

| Species | Autumn | Winter | Spring | Summer | TOTAL |
| --- | --- | --- | --- | --- | --- |
| Crimson rosella *(Platycercus elegans)* | 15 | 30 | 9 | 3 | 57 |
| Galah *(Eolophus roseicapillus)* | 6 | 5 | 18 | 2 | 31 |
| Sulphur-crested cockatoo *(Cacatua galerita)* | 3 | 15 | 2 | 1 | 21 |
| Blue-winged parrot *(Neophema chrysostoma)* | 0 | 0 | 17 | 0 | 17 |
| Eastern rosella *(Platycercus eximius)* | 3 | 0 | 0 | 0 | 3 |
| Rainbow lorikeet *(Trichoglossus moluccanus)* | 0 | 2 | 0 | 0 | 2 |
| Red-rumped parrot *(Psephotus haematonotus)* | 0 | 0 | 1 | 0 | 1 |
| TOTAL | 27 | 52 | 47 | 6 | 132 |

**Supplementary Table S3** Results from BLASTn analysis for 16S rRNA nucleotide sequences and partial *ompA* sequences obtained in this study, as determined using the non-redundant nucleotide (nr/nt) database and 16S ribosomal RNA sequence database (for 16S).

| Sample  /host | Size (bp) | Gene | GenBank accession number | nr/nt database | | | | 16S bacteria/archaea database | | | |
| --- | --- | --- | --- | --- | --- | --- | --- | --- | --- | --- | --- |
|  |  |  |  | Identity/top  BLAST hit | Sequence identity | Query cover | E value | Identity | Sequence identity | Query cover | E value |
| 5015/  crimson rosella | 739 | *ompA* | MT875197 | *Chlamydia psittaci* (genotype A) | 100% | 100% | 1e-  119 | n/a | | | |
| 5027/galah | 711 | *ompA* | MT875198 | *Chlamydia psittaci* (genotype A) | 99-100% | 100% | 1e-119 | n/a | | | |
| 5026/galah | 195 | 16S | MT872005 | *Chlamydia psittaci* | 100% | 100% | 9e-96 | *Chlamydia psittaci* 6BC 16S ribosomal RNA | 100% | 100% | 1e-99 |
| 5102/  crimson rosella | 168 | 16S | MT872000 | *Chlamydia psittaci* | 99% | 100% | 4e-79 | *Chlamydia psittaci* 6BC 16S ribosomal RNA | 99% | 100% | 4e-83 |
| 5457/  sulphur-crested cockatoo | 212 | 16S | MT889682 | *Chlamydia psittaci* | 99-100% | 100% | 23-103 | *Chlamydia psittaci* 6BC 16S ribosomal RNA | 99-100% | 100% | 2e-107 |
| 5051/  crimson rosella | 213 | 16S | MT889721 | *Chlamydia psittaci* | 100% | 100% | 1e-105 | *Chlamydia psittaci* 6BC 16S ribosomal RNA | 100% | 100% | 1e-109 |
| 5137/  crimson rosella | 281 | 16S | MT889690 | Uncultured Parachlamydiaceae bacterium, clone 5134 16S ribosomal RNA gene, partial sequence (MT356623) | 100% | 84% | 2e-119 | Neochlamydia hartmannellae strain A1Hsp 16S ribosomal RNA, partial sequence (NR_025037) | 88% | 100% | 2e-93 |
| 5066/  crimson rosella | 222 | 16S | MT889722 | *Chlamydia psittaci* | 100% | 100% | 1e-110 | *Chlamydia psittaci* 6BC 16S ribosomal RNA | 100% | 100% | 1e-114 |

**
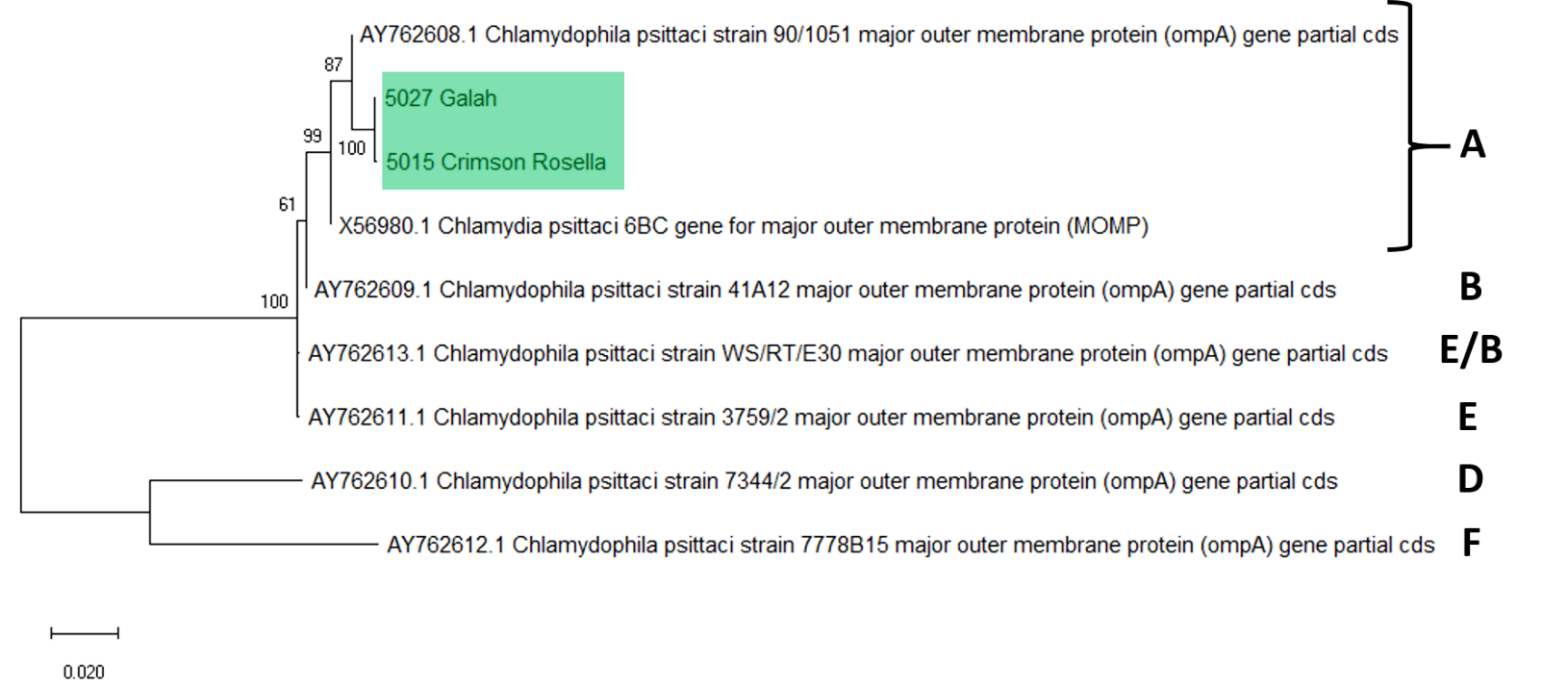
**

**Supplementary Figure S1** Neighbour-join dendrogram based on analysis of the partial (700-750bp) *ompA* sequence obtained using the reverse CTU/CTL primer. Sequences were aligned in MEGA-X using ClustalW, and the dendrogram was creating using the neighbour-joining method with 1000 bootstrap repetitions. Samples obtained in this study are highlighted in green, and *C. psittaci* genotype is indicated on the right.

**Supplementary Table S4** Level of significance (*p-*value) for each pairwise comparison in seroprevalence between species, derived from post-hoc Tukey’s tests. *denotes comparisons where species significantly differ in seroprevalence (where *p* < 0.05).

| Species | Crimson rosella | Galah | Sulphur-crested cockatoo | Blue-winged parrot |
| --- | --- | --- | --- | --- |
| Crimson rosella |  | <0.001* | 0.802 | 0.997 |
| Galah | <0.001* |  | 0.013* | 0.059 |
| Sulphur-crested cockatoo | 0.802 | 0.013* |  | 0.991 |
| Blue-winged parrot | 0.997 | 0.059 | 0.991 |  |

**Supplementary Table S5** GLM testing for an effect of capture time on *Chlamydiales* and *C. psittaci* PCR prevalence, for all focal species, and for crimson rosellas separately. *denotes significant terms (where *p* < 0.05).

| Cohort tested | Response | Predictor | *χ*^2^ | *df* | *p-* value |
| --- | --- | --- | --- | --- | --- |
| All focal species (n=117) | Chlamydiales PCR prevalence | Sex | 1.543 | 1 | 0.210 |
|  |  | Species | 13.16 | 3 | 0.004* |
|  |  | Field site | 0.030 | 1 | 0.862 |
|  |  | Season | 3.959 | 3 | 0.266 |
|  |  | Time caught | 0.905 | 1 | 0.341 |
|  | *C. psittaci* PCR prevalence | Sex | 1.620 | 1 | 0.203 |
|  |  | Species | 0.964 | 3 | 0.810 |
|  |  | Field site | 0.320 | 1 | 0.571 |
|  |  | Season | 1.012 | 3 | 0.798 |
|  |  | Time caught | 1.620 | 1 | 0.203 |
| Crimson rosellas only (n=51) | Chlamydiales PCR prevalence | Sex | 0.724 | 1 | 0.395 |
|  |  | Field site | 0.031 | 1 | 0.860 |
|  |  | Season | 3.404 | 3 | 0.333 |
|  |  | Time caught | 0.132 | 1 | 0.717 |
|  | *C. psittaci* PCR prevalence | Sex | 1.917 | 1 | 0.166 |
|  |  | Field site | 0.019 | 1 | 0.889 |
|  |  | Season | 0.989 | 3 | 0.811 |
|  |  | Time caught | 3.883 | 1 | 0.049* |

**Supplementary Table S6** Associations between *Chlamydiales* PCR prevalence, *C. psittaci* PCR prevalence and seroprevalence with body mass and packed cell volume (PCV). *denotes significant terms (where *p* < 0.05)

| Response | Predictor | | *χ*^2^ | | *df* | *p-* value |
| --- | --- | --- | --- | --- | --- | --- |
| Body mass (n=98) | Sex | 12.66 | | 1 | | <0.001* |
|  | Species | 2216.6 | | 2 | | <0.001* |
|  | *Chlamydiales* | 2.32 | | 1 | | 0.131 |
| Body mass (n=98) | Sex | 13.30 | | 1 | | <0.001* |
|  | Species | 2173.7 | | 2 | | <0.001* |
|  | *C. psittaci* | 0.618 | | 1 | | 0.434 |
| Body mass (n=103) | Sex | 13.304 | | 1 | | <0.001* |
|  | Species | 1639.5 | | 3 | | <0.001* |
|  | Seroprevalence | 3.92 | | 1 | | 0.051 |
| PCV (n=90) | Sex | 0.030 | | 1 | | 0.862 |
|  | Species | 6.966 | | 2 | | 0.002* |
|  | *Chlamydiales* | 3.248 | | 1 | | 0.076 |
| PCV (n=90) | Sex | 0.001 | | 1 | | 0.975 |
|  | Species | 6.076 | | 2 | | 0.003* |
|  | *C. psittaci* | 0.855 | | 1 | | 0.358 |
| PCV (n=93) | Sex | 0.352 | | 1 | | 0.555 |
|  | Species | 8.373 | | 3 | | <0.001* |
|  | Seroprevalence | 3.346 | | 1 | | 0.071 |

**Supplementary Table S7** Associations between *Chlamydiales* PCR prevalence, *C. psittaci* PCR prevalence and seroprevalence with residual body mass for crimson rosellas and galahs. Residual body mass was calculated separately by host species. *denotes significant terms (where *p* < 0.05)

| Species | Response | Predictor | *χ*^2^ | | *df* | *p* - value |
| --- | --- | --- | --- | --- | --- | --- |
| Crimson rosella | Residual body mass (n=42) | Sex  Chlamydiales | 15.98 | 1 | | <0.001* |
|  |  |  | 2.499 | 1 | | 0.114 |
| Crimson rosella | Residual body mass (n=42) | Sex | 16.267 | 1 | | <0.001* |
|  |  | *C*. *psittaci* | 1.305 | 1 | | 0.253 |
| Crimson rosella | Residual body mass (n=43) | Sex | 15.344 | 1 | | <0.001* |
|  |  | Seroprevalence | 0.294 | 1 | | 0.588 |
| Galah | Residual body mass (n=8) | Sex | 2.810 | 1 | | 0.094 |
|  |  | Chlamydiales | 0.072 | 1 | | 0.789 |
| Galah | Residual body mass (n=8) | Sex | 2.387 | 1 | | 0.122 |
|  |  | *C. psittaci* | 0.205 | 1 | | 0.651 |
| Galah | Residual body mass (n=8) | Sex | 2.674 | 1 | | 0.102 |
|  |  | Seroprevalence | 0.027 | 1 | | 0.870 |

**Supplementary Table S8** Level of significance (*p-*value) for each pairwise comparison in packed cell volume (PCV) between species, derived from post-hoc Tukey’s tests. *denotes comparisons where species significantly differ in prevalence (where *p* < 0.05)

| Species | Mean PCV (±SD) | Crimson rosella | Galah | Sulphur-crested cockatoo | Blue-winged parrot |
| --- | --- | --- | --- | --- | --- |
| Crimson rosella | 0.56 (±0.05) |  | 0.001* | 0.984 | 0.005* |
| Galah | 0.59 (±0.04) | <0.001* |  | 0.032* | 0.973 |
| Sulphur-crested cockatoo | 0.56 (±0.03) | 0.984 | 0.032* |  | 0.039* |
| Blue-winged parrot | 0.60 (±0.03) | 0.004* | 0.998 | 0.039* |  |

**Supplementary Table S9** Proportion of capture events (n=102) with each combination of *Chlamydia* genus PCR and seroprevalence assay results. *Chlamydiales* positive samples where the bacterial species was unknown (n=36) were excluded from this analysis.

| PCR and sequencing outcome | ImmunoComb® outcome | No. of individuals | Percentage of capture events (%) |
| --- | --- | --- | --- |
| + | + | 6 | 5.9 |
| + | - | 10 | 9.8 |
| - | + | 25 | 24.5 |
| - | - | 61 | 59.8 |

**Supplementary Table S10** Association between PCR prevalence and seroprevalence (n=102). *Chlamydiales* positive samples where the bacterial species was unknown (n=36) were excluded.

| Predictive PCR prevalence | Odds ratio | s.e. | Lower 95% CI | Upper 95% CI | *p*-value |
| --- | --- | --- | --- | --- | --- |
| *C. psittaci* (positive) | 0.926 | 0.588 | 0.269 | 2.826 | 0.895 |
| *Chlamydia* genus (positive) | 1.154 | 0.561 | 0.364 | 3.387 | 0.799 |
| *Chlamydiales* (positive) | 0.952 | 0.518 | 0.328 | 2.569 | 0.925 |

`

**No. of days since initial capture**

**PCR -**

**Chlamydiales PCR status**

**PCR +**

**Supplementary Figure S2** Changes in *Chlamydiales* PCR status over time for recaptured individuals. Seroprevalence status of each individual did not change between capture events; dotted lines indicate birds which were seropositive and solid lines indicate birds which were seronegative. Black data points are crimson rosellas*,* red data points are galahs, and blue data points are eastern rosellas.

**Supplementary Table S11** Association between *Chlamydiales* PCR status on first capture and *Chlamydiales* status and seroprevalence status on recapture, in crimson rosellas (n=29).

| Response | Predictor | Odds ratio | s.e. | *z* | *p*-value |
| --- | --- | --- | --- | --- | --- |
| *Chlamydiales* status at recapture (+/-) | *Chlamydiales* status at initial capture (+/-) | 20.733 | 0.979 | -0.317 | 0.751 |
| Seroprevalence status at recapture (+/-) | *Chlamydiales* status at initial capture (+/-) | 0.200 | 1.659 | -0.970 | 0.332 |
